# Identification of a Novel Protein Arginine Methyltransferase 5 Inhibitor in Non-small Cell Lung Cancer by Structure-Based Virtual Screening

**DOI:** 10.3389/fphar.2018.00173

**Published:** 2018-03-01

**Authors:** Qianqian Wang, Jiahui Xu, Ying Li, Jumin Huang, Zebo Jiang, Yuwei Wang, Liang Liu, Elaine Lai Han Leung, Xiaojun Yao

**Affiliations:** ^1^State Key Laboratory of Quality Research in Chinese Medicine, Macau Institute for Applied Research in Medicine and Health, Macau University of Science and Technology, Taipa, Macau; ^2^State Key Laboratory of Respiratory Diseases, Guangzhou Institute of Respiratory Disease, The First Affiliated Hospital of Guangzhou Medical College, Guangzhou, China; ^3^Department of Respiratory Medicine, Taihe Hospital, Hubei University of Medicine, Hubei, China; ^4^State Key Laboratory of Applied Organic Chemistry, Department of Chemistry, Lanzhou University, Lanzhou, China

**Keywords:** protein arginine methyltransferase 5, non-small cell lung cancer, T1551, virtual screening, molecular dynamics simulation

## Abstract

Protein arginine methyltransferase 5 (PRMT5) is able to regulate gene transcription by catalyzing the symmetrical dimethylation of arginine residue of histone, which plays a key role in tumorigenesis. Many efforts have been taken in discovering small-molecular inhibitors against PRMT5, but very few were reported and most of them were SAM-competitive. EPZ015666 is a recently reported PRMT5 inhibitor with a new binding site, which is different from S-adenosylmethionine (SAM)-binding pocket. This new binding site provides a new clue for the design and discovery of potent and specific PRMT5 inhibitors. In this study, the structure-based virtual screening targeting this site was firstly performed to identify potential PRMT5 inhibitors. Then, the bioactivity of the candidate compound was studied. MTT results showed that compound T1551 decreased cell viability of A549 and H460 non-small cell lung cancer cell lines. By inhibiting the methyltransferase activity of PRMT5, T1551 reduced the global level of H4R3 symmetric dimethylation (H4R3me2s). T1551 also downregulated the expression of oncogene FGFR3 and eIF4E, and disturbed the activation of related PI3K/AKT/mTOR and ERK signaling in A549 cell. Finally, we investigated the conformational spaces and identified collective motions important for description of T1551/PRMT5 complex by using molecular dynamics simulation and normal mode analysis methods. This study provides a novel non-SAM-competitive hit compound for developing small molecules targeting PRMT5 in non-small cell lung cancer.

## Introduction

Protein arginine methyltransferases (PRMTs) are a class of enzymes that transfer a methyl group from the cofactor S-adenosylmethionine (SAM) to arginine omega nitrogen of substrate protein. Based on product specificity, PRMTs can be divided into three subclasses: type I, II, and III, which asymmetrically dimethylate, symmetrically dimethylate, and monomethylate their substrates, respectively (Bedford and Clarke, 2009). Protein arginine methyltransferase 5 (PRMT5), as a type II PRMT, is responsible for catalyzing the symmetrical dimethylation of arginine residue of substrate proteins, which has been implicated in diverse cellular and biological processes including transcriptional regulation, RNA metabolism and ribosome biogenesis (Liu et al., 2011; Shilo et al., 2013; Wei et al., 2013; Yang and Bedford, 2013; Deuker and McMahon, 2014; Stopa et al., 2015). An increasing number of studies emphasized that PRMT5 was upregulated in lymphomas, breast cancer, lung cancer, colorectal cancer, and glioblastoma (Ibrahim et al., 2014; Yan et al., 2014; Li et al., 2015; Sheng and Wang, 2016). For instance, Ibrahim et al. (2014) demonstrated that a high cytoplasmic expression of PRMT5 was closely related to high-grade subtypes of primary lung adenocarcinomas and a poor prognosis. Sheng and Wang (2016) pointed out that PRMT5 could regulate multiple signaling pathways to promote lung cancer cell proliferation. All of these suggest that PRMT5 is a promising therapeutic target in lung cancer. However, although many efforts have been made in discovering PRMT5 inhibitors, very few were reported (Alinari et al., 2015; Smil et al., 2015; Mao et al., 2017), and they either occupied SAM-binding site or mimicked SAM. Recently, EPZ015666 has been shown to exhibit remarkably antitumor activity by inhibiting PRMT5, and the pre-clinical studies have also showed that both cell lines and xenograft models of mantle cell lymphoma were sensitive to EPZ015666 (Chan-Penebre et al., 2015). Importantly, the resolved PRMT5-SAM-EPZ015666 crystal complex shows that EPZ015666 does not compete with SAM, but locates in a new pocket (different from SAM-binding site) of PRMT5. This binding site in PRMT5 provides us a new way to discovery and development of more potent and specific PRMT5 inhibitors.

Structure-based virtual screening using molecular docking has become a powerful tool in the drug discovery for rapidly enriching hits from large pools of compound databases. Nowadays, it has been successfully applied to discover novel inhibitors of epigenetic targets, such as SET7, KDM4B, and SIRT2 (Chu et al., 2014; Meng et al., 2015; Huang et al., 2017). The successful use of structure-based virtual screening in the above mentioned epigenetic targets inspires us to identify the novel inhibitor against the non-SAM-binding site of PRMT5. The activity of the identified inhibitors will be further studied on their effects of the biological functions of cancer cells, histone substrate methylation, target gene expression and related signaling pathway. Here, 158 candidate compounds were firstly obtained by the structure-based virtual screening method. MTT assay results showed that among them T1551 had strongest cytotoxicity on A549 non-small cell lung cancer cell line. In addition to inhibiting PRMT5 methyltransferase activity, a series of functional assays showed that T1551 reduced symmetric dimethylation level of H4R3, downregulated the protein expressions of two target genes of PRMT5, FGFR3, and eIF4E, and inhibited the activation of PI3K/AKT/mTOR and ERK signaling. Finally, molecular dynamics simulations and normal mode analysis were performed to study the detailed binding mode and conformational space of T1551/PRMT5 complex. The identification of this novel PRMT5 inhibitor T1551 and its inhibitory mechanism study will be helpful for the development of PRMT5-targeting cancer treatment.

## Materials and Methods

### Molecular Docking-Based Virtual Screening

Molecular docking-based virtual screening was carried out with Schrödinger software package (Schrödinger, LLC, New York, NY, United States; Schrödinger, 2015). The crystal structure of PRMT5 complexed with cofactor SAM and inhibitor EPZ015666 was derived from Protein Data Bank (PDB ID: 4X61). The protein was first prepared in Protein Preparation Wizard module, including adding hydrogens, refining loop region and minimization. Grid box was generated on the size and center of EPZ015666. Previously, SAM was proved to form crucial cation–π interactions with EPZ015666 and contribute to the binding affinity of PRMT5 inhibitors (Chan-Penebre et al., 2015). Here, to test the role of SAM in docking, enrichment factors (EFs) of virtual screening for PRMT5-EPZ015666 with and without SAM were calculated and compared. Firstly, 16 active derivatives of EPZ015666 were collected from the published paper (Duncan et al., 2015). Eight hundred decoys were then generated at a ratio of 1:50 with DUD-E (Mysinger et al., 2012). All the actives and decoys were docked into EPZ015666 binding site of PRMT5 with and without SAM, respectively. Finally, the 1 and 10% EFs for PRMT5-EPZ015666 and PRMT5-EPZ015666-SAM models were calculated, respectively. For the ligands, prior to virtual screening, a total of 1,671,908 compounds from Chemdiv, Specs and TargetMol databases were filtered by pan-assay interference structures (PAINS) (Baell and Holloway, 2010) and “Lipinski’s rule of five” to remove those with false positivity, function group and poor absorption/permeability. Then, the obtained compounds were prepared with Ligand Preparation module. Three-level (HTVS, SP, and XP) molecular docking-based virtual screening was successively performed using Glide module. The top 10% (1,706) compounds ranked by glide score were clustered into 200 groups. By visually inspecting the binding poses of PRMT5-inhibitor, 158 compounds were selected for experimental validation. All compounds were purchased from Topscience company (Shanghai, China).

### Cell Culture and Cytotoxicity Assay

A549 and H460 cells (two non-small cell lung cancer cell lines) were purchased from ATCC, cultivated in RPMI 1640 medium supplemented with 10% FBS (Gibco Products, Big Cabin, OK, United States), 1% penicillin-streptomycin solution, and maintained at 37°C in a CO_2_ incubator with 5% CO_2_. One hundred and fifty-eight compounds from virtual screening were dissolved in DMSO and stored at -40°C. To rapidly identify the compounds with strong inhibitory activity, 20 μM concentration for each compound was firstly used to treat A549 cell line for 72 h. During the MTT assay test, cells were firstly seeded on a 96-well microplate with 3,000 cells/well, cultured overnight for cell adhesion, and treated with DMSO (10.0 μM) or various concentrations (2.5, 5.0, and 10.0 μM) of the studied compound for 24, 48, and 72 h. Then, each well was added 10 μL MTT (5 mg/mL) and incubated for 4 h at 3°C, followed by adding 100 μL acidic isopropanol (10% SDS and 0.01 mol/L HCl). Finally, the absorbance at 570 nm was measured by a Microplate Reader (Tecan US, Inc., Morrisville, NC, United States). Cell viability was calculated relative to untreated controls, and the results were based on at least three independent experiments.

### *In Vitro* Enzymatic Assays

PRMT5 enzymatic assay was carried out by Shanghai ChemPartner Company (998 Halei Road, Pudong New Area, Shanghai, 201203, China), as did previously by Ji et al. (2017). To obtain the specific IC_50_ value, T1551 was diluted into 10 concentrations. PRMT5 protein was purchased from BPS bioscience (Cat. No. 51045), and SAM/SAH were purchased from Sigma. Inc. (Cat. No. A7007-100MG and No. A9384-25MG). T1551 was prepared as 10 mM stock in DMSO and diluted to the final concentration in DMSO. PRMT5 and substrates were incubated with indicated concentrations of T1551 in a 384-well plate for 60 min at room temperature. Then, acceptor and donor solutions were added to label the residual substrates of PRMT5. The labeling process was lasting for 60 min at room temperature, followed by reading endpoint with EnSpire with Alpha mode. In the *in vitro* enzymatic assays, 1% DMSO was used as vehicle control for normalization.

### Western Blot Analysis

Cells were washed twice with cold PBS, and lysed in RIPA lysis buffer containing protease and phosphatase inhibitors to extract total protein. Cell lysates were centrifuged for 5 min (12,000 *g*, 4°C), and the supernatant was collected. Protein concentrations were determined by Bio-Rad protein Assay kit (Bio-Rad, Philadelphia, PA, United States). Equal amounts of protein (50 μg) were separated on a 10% SDS–PAGE gel, and transferred to a nitrocellulose (NC) membrane at 300 mA and 4°C for 1 h. The membrane was incubated with primary antibody (1:1000), and then with a fluorescence-conjugated secondary antibody (1:10000). The primary antibody against PRMT5 was purchased from Merck Millipore Ltd., (Germany); antibodies against H4R3me2s and H4 were purchased from Abcam (Cambridge, MA, United States); antibodies against FGFR3 and eIF4E were purchased from Santa Cruz Biotechnology (Dallas, TX, United States); antibodies against total/phospho-AKT, total/phospho-ERK and total/phospho-mTOR were purchased from Cell Signaling Technology (Danvers, MA, United States). GAPDH was used as the loading control and for normalization. The signal intensity of the membranes was detected with a LI-COR Odyssey Scanner (Belfast, ME, United States).

### Molecular Dynamics Simulation

To reveal the interaction features of T1551 and PRMT5, molecular dynamics (MD) simulations were used for sampling the conformational spaces of PRMT5-T1551 complex. Normal mode analysis was used for identifying important collective motions for the complex. All MD simulations were performed with Amber 16 software (Case et al., 2017). The Amber ff14SB force field (Maier et al., 2015) was used for PRMT5, and general amber force field (Wang et al., 2004) was utilized to parameterize inhibitors with their charges assigned by restrained electrostatic potential partial charges. TIP3P water was used to solvate the complex systems, with the solute 12 Å away from water box boundary. Chloride ions were added to neutralize the system. Then, 150 mM NaCl was added to mimic the physiological conditions. After minimization, heating and equilibration, 100 ns production run was carried out without any restraints in NPT ensemble. System temperature and pressure were regulated with Langevin thermostat and Berendsen barostat, respectively. All the bonds involving hydrogen were constrained by SHAKE algorithm allowing an integration time step of 2 fs. Particle mesh Ewald method (Linse and Linse, 2014) was used to calculate long-range electrostatic interactions. The binding free energy of inhibitors and PRMT5 was calculated by molecular mechanics generalized-born surface area (MM-GBSA) method (Hou et al., 2010; Platania et al., 2015; Wang et al., 2017). A single trajectory and three time-frames protocols were adopted here. Specifically, a total of 500 snapshots were extracted from the last 10, 20, and 40 ns trajectory, respectively. The normal mode analysis was performed to identify the collection motions of PRMT5-inhibitor complex during MD simulation, by using cpptraj in Amber 16 and Normal Mode Wizard plugin in VMD 1.9.

### Statistical Analysis

Descriptive analytical data were presented as mean ± SEM. Multiple comparisons were evaluated by one-way analysis of variance (ANOVA) using Graph Prim 5.0. *P* < 0.05 was considered statistically significant.

## Results

### The Selection of Candidate Compounds by Virtual Screening

In this study, we aim to find the non-SAM mimics, so EPZ015666-binding site, not SAM-binding site, was targeted in our virtual screening. Enrichment factor calculations showed that the 1 and 10% EFs for PRMT5-EPZ015666-SAM model were 44.6 and 8.7, higher than that (38.3 and 6.8) for PRMT5-EPZ015666 model. The area under receiver operating characteristic curve (AUC) for the former (0.96) was also higher than that for the latter (0.92). Both of two parameters suggested that SAM was helpful for enriching active compounds in the compound library. Therefore, SAM was remained as a part of the receptor in the screening.

By three-level (HTVS, SP, and XP) screenings, the top-1706 compounds ranked by glide score were remained and then clustered into 200 groups using *k*-means clustering protocol integrated in Canvas 2.4. When selecting the candidate compounds, the following criteria was considered: (1) choosing one compound at most in a group to retain structural diversity; (2) occupying the binding pocket with molecular size neither too big nor too small; (3) choosing the one with smaller molecular weight or/and lower MM/GBSA score if compounds are similar; (4) forming the reported interactions with the key residues of PRMT5 (Chan-Penebre et al., 2015). For instance, Phe327 forms π–π interactions with THIQ ring of EPZ015666; THIQ forms cation–π interactions with methyl group of SAM; EPZ015666 interacts with the backbone -NH of Phe580 and side chains of Glu444. Based on these, 158 candidates were selected and purchased at last.

### T1551 Decreases Cell Viability of A549 Cell

**FIGURE 1:**
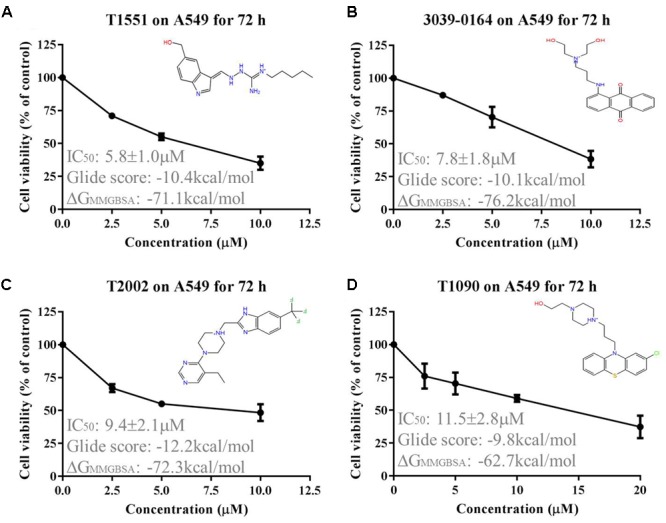
Cytotoxic effects of **(A)** T1551, **(B)** 3039-0164, **(C)** T2002, and **(D)** T1090 on A549 cell, as analyzed by MTT assay. A549 cell was treated with each inhibitor for 72 h, respectively. Results were presented as mean ± SEM (*n* = 4). Glide score represented the docking score of inhibitor and PRMT5, and ΔG_MMGBSA_ represented the post-docking rescore of inhibitor and PRMT5.

**FIGURE 2:**
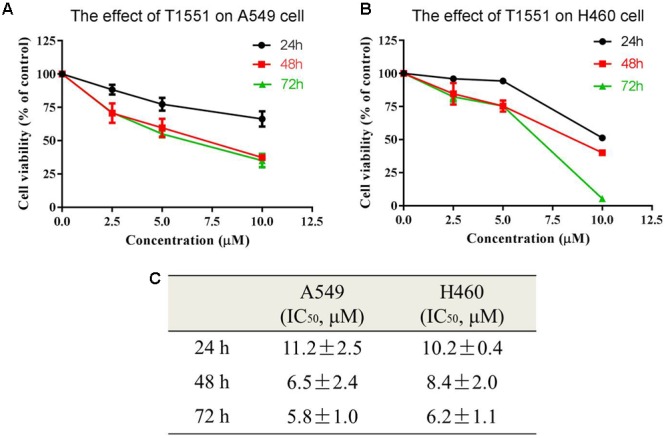
Cytotoxic effects of T1551 on **(A)** A549 and **(B)** H460 cells by MTT assay. **(C)** IC_50_ values of T1551 on A549 and H460 cell lines. Cells were treated with each inhibitor for 24, 48, and 72 h, respectively. Data was presented as mean ± SEM (*n* = 4).

The obtained 158 candidate compounds were then tested for MTT assay to determine their inhibitory activity. Many recent studies have showed that PRMT5 is upregulated in A549 non-small cell lung cancer cell line (Gu et al., 2012; Wei et al., 2012; Lim et al., 2014). A549 cell line was thus used here. To rapidly identify the compounds with the strong inhibitory activity, 20 μM concentration for each compound was firstly used to treat A549 cell for 72 h. The result showed that among 158 compounds there were four compounds exhibiting the >50% inhibitory percentage on A549 cell at 20 μM. Since T1551 had the strongest inhibitory activity (72 h, 50% inhibition concentration IC_50_ = 5.8 ± 1.0 μM) (**Figure 1**) and was chosen as the hit, a range of T1551 concentrations (0, 2.5, 5.0, and 10.0 μM) for 24, 48, and 72 h were then used to treat A549 to calculate its IC_50_ values. As shown in **Figure 2**, T1551 exhibited significant anti-proliferation on A549 cell at 24 h in a concentration-dependent manner, with the IC_50_ value of 11.2 ± 2.5 μM. The cytotoxic effects of T1551 were also verified using H460 cell, another NSCLC cell line with PRMT5 overexpression (**Figures 2B,C**).

### T1551 Inhibits PRMT5 Methyltransferase Activity and Decreases Symmetric Dimethylation Level of Histone 4

**FIGURE 3:**
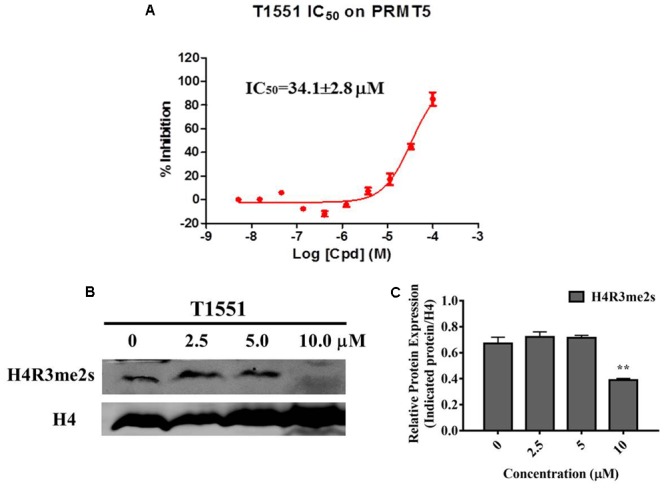
**(A)** Inhibition of T1551 on PRMT5 methyltransferase activity. **(B)** Protein expression levels of H4R3me2s in A549 cell treated with T1551 at different concentrations (0, 2.5, 5.0, and 10.0 μM). **(C)** Densitometric analysis of band intensities of H4R3me2s. Western blot analysis was performed for 24 h, with at least three independent experiments. Data was presented as mean ± SEM (*n* = 3), with ^∗∗^*p* < 0.01 for comparison between control group (DMSO-treated group) and T1551-treated group.

AlphaLISA assay was carried out to investigate the influence of T1551 on enzymatic activity of PRMT5. As shown in **Figure 3A**, T1551 inhibited PRMT5 enzyme activity in a dose-dependent manner. The corresponding IC_50_ value was 34.1 ± 2.8 μM, suggesting that T1551 directly inhibited the methyltransferase function of PRMT5. PRMT5-driven methylation of arginine residues can lead to symmetric dimethylation of arginine residue 3 of histone 4 (H4R3me2s), which in turn alters chromatin structure to promote transcriptional repression (Branscombe et al., 2001; Zhao et al., 2009; Chen et al., 2017). To investigate the effect of T1551 on PRMT5 catalytic substrate, we measured the expression level of H4R3me2s protein with and without T1551 in A549 cell. The total H4 was used as loading control. From **Figures 3B,C**, we observed that after the treatment with T1551 for 24 h, the global level of H4R3me2s was notably decreased. Therefore, from the perspective of histone substrate, T1551 indeed inhibited the catalytic ability of PRMT5 methyltransferase.

### T1551 Downregulates the Expression of PRMT5 Target Genes

**FIGURE 4:**
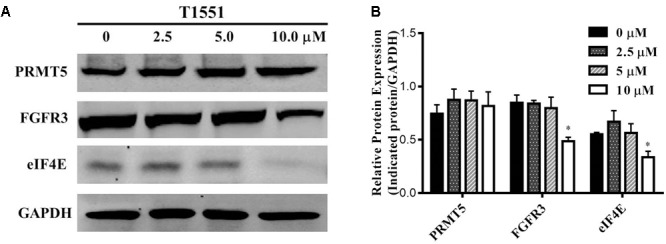
**(A)** Protein expression levels of oncogene FGFR3 and eIF4E in A549 cell treated with T1551 at different concentrations (0, 2.5, 5.0, and 10.0 μM). **(B)** Densitometric analysis of band intensities of PRMT5, FGFR3, and eIF4E proteins. Western blot analysis was performed for 24 h, with at least three independent experiments. Data was presented as mean ± SEM (*n* = 3), with ^∗^*p* < 0.05 for comparison between control group (DMSO-treated group) and T1551-treated group.

PRMT5 exerts its function by regulating the expression of target genes, such as oncogene FGFR3 and eIF4E (Zhang et al., 2015). FGFR3 and eIF4E were previously reported to frequently overexpress in lung cancer, myeloma, and ovarian cancers (van Rhijn et al., 2001; De Benedetti and Graff, 2004; Culjkovic-Kraljacic et al., 2012), thus playing an important role in tumor occurrence and development. Especially, according to several studies (Desai and Adjei, 2016; Babina and Turner, 2017) recently published, FGFR signaling has been considered as a promising target for lung cancer therapy. As can be seen from **Figure 4**, FGFR3 and eIF4E expressions were significantly decreased in A549 cell treated with 10.0 μM T1551. This reflects that T1551 may reduce FGFR3 and eIF4E expression by inhibiting PRMT5.

### T1551 Suppresses the Activation of AKT, ERK, and mTOR

As mentioned above, FGFR3 signaling is an important target for lung cancer treatment. In this FGFR3 pathway, PRMT5 participates in regulating FGFR3 downstream targets such as AKT, ERK, and mTOR (Wei et al., 2012). From the previous study, silencing PRMT5 could reduce FGFR3 expression, leading to the repression of AKT and ERK and subsequent inhibition of mTOR through AKT/mTOR or ERK pathway (Zhang et al., 2015).

**FIGURE 5:**
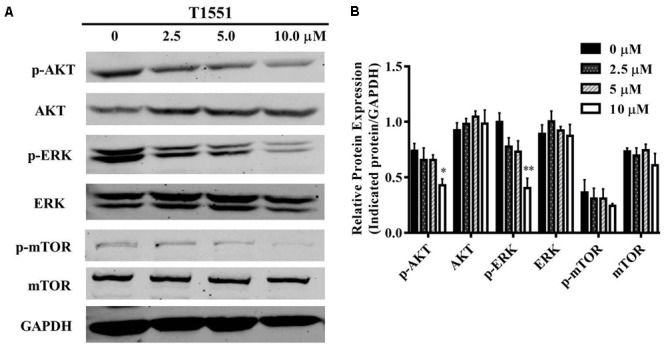
**(A)** Protein expression levels of p/T-AKT, p/T-ERK, and p/T-mTOR in A549 cell treated with T1551 at different concentrations (0, 2.5, 5.0, and 10.0 μM). **(B)** Densitometric analysis of band intensities of p/T-AKT, p/T-ERK, and p/T-mTOR. Western blot analysis was performed for 24 h, with at least three independent experiments. Data was presented as mean ± SEM (*n* = 3), with ^∗^*p* < 0.05 and ^∗∗^*p* < 0.01 for comparison between control group (DMSO-treated group) and T1551-treated group.

To gain further insight into the molecular mechanism underlying PRMT5-dependent regulation of FGFR3, we examined whether T1551 could regulate the activation of AKT, ERK, and mTOR through inhibiting PRMT5. From **Figures 5A,B**, we observed that the protein levels of phosphorylated AKT and ERK were significantly reduced, especially at the 10 μM T1551 concentration, implying that T1551 suppressed the activation of PI3K/AKT/mTOR and ERK signaling mediated by PRMT5.

### Inhibition Mechanism of T1551 Inhibitor for PRMT5 Protein

**FIGURE 6:**
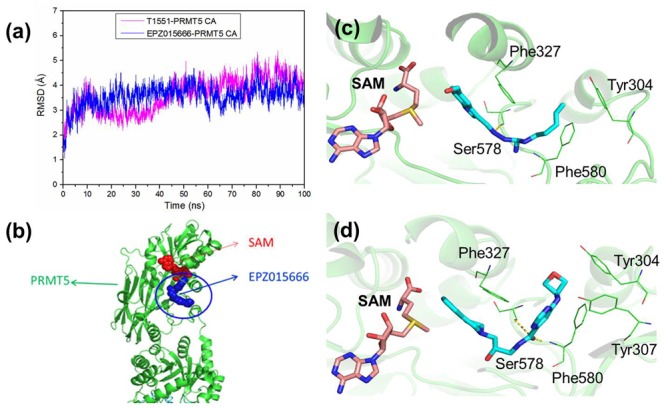
**(a)** Time series of RMSDs of protein CA atoms during the 100 ns simulation in PRMT5-SAM-T1551 and PRMT5-SAM-EPZ015666 systems. **(b)** Crystal structure of PRMT5-SAM-EPZ015666 (PDBID: 4X61). The binding modes of PRMT5 with **(c)** T1551 and **(d)** EPZ015666. Both two complex structures were extracted from the last equilibrated 20 ns trajectory by clustering analysis.

To investigate the detailed binding modes of PRMT5-inhibitors and compare the interaction features of T1551 and EPZ015666 with PRMT5, a single 100 ns MD simulations for PRMT5-SAM-T1551 and PRMT5-SAM-EPZ015666 systems were performed, respectively. Based on the obtained trajectory, with respect to the initial structure, the root-mean-square deviations (RMSDs) of protein CA atoms in PRMT5-SAM-T1551 and PRMT5-SAM-EPZ015666 systems were monitored to assess the overall stability of simulations. From **Figure 6a**, RMSDs of each system almost remained stable from 60 ns, indicating the convergence of the simulated trajectory. By calculating the binding free energies of PRMT5 with T1551 and EPZ015666, we can identify the energy origin of inhibitors binding to PRMT5. Here, considering the large size of PRMT5 and inhibitor complex (more than 600 residues, **Figure 6b**), entropic contribution was neglected. The predicted ΔG_GB_ for PRMT5-T1551 was higher than that of PRMT5-EPZ015666 (e.g., -32.11 ± 0.14 vs. -40.09 ± 0.18 kcal/mol in last 10 ns) in three replicas, exhibiting a consistent ranking with experimental results (**Table 1**; Chan-Penebre et al., 2015). Among the individual energy parts, van der Waals interaction (ΔE_vdw_) predominated the total energy in two systems, while non-polar solvation part (ΔG_sol_np_GB_) contributed marginally to inhibitor binding. Therefore, the energetic origin of T1551/EPZ015666 inhibiting PRMT5 is mainly derived from Δ*E*_vdw_.

**Table 1:** The calculated binding free energy and its components (kcal/mol) of PRMT5 with T1551 and EPZ015666 complexes based on the last 10, 20, and 40 ns MD trajectory.

	Δ*E*_vdw_	Δ*E*_ele_	Δ*G*_sol_np_GB_	Δ*G*_sol_polar_GB_	Δ*G*_GB_
**PRMT5-T1551**					
Last 10 ns	–40.98 ± 0.13	–29.73 ± 0.18	–5.49 ± 0.01	44.08 ± 0.13	–32.11 ± 0.14
Last 20 ns	–41.69 ± 0.12	–29.89 ± 0.16	–5.57 ± 0.01	44.64 ± 0.12	–32.50 ± 0.14
Last 40 ns	–41.80 ± 0.12	–30.89 ± 0.19	–5.65 ± 0.01	45.32 ± 0.11	–33.02 ± 0.15
**PRMT5-EPZ015666**					
Last 10 ns	–49.72 ± 0.15	–43.09 ± 0.50	–6.96 ± 0.01	59.67 ± 0.39	–40.09 ± 0.18
Last 20 ns	–48.98 ± 0.15	–43.72 ± 0.48	–6.94 ± 0.01	58.51 ± 0.38	–41.13 ± 0.17
Last 40 ns	–49.66 ± 0.17	–49.51 ± 0.59	–7.12 ± 0.01	64.17 ± 0.46	–42.12 ± 0.19

*ΔG was estimated from gas-phase energy and solvation free energy. The former contains an electrostatic term (ΔE_*ele*_) and a van der Waals term (ΔE_*vdw*_). The latter is decomposed into polar (ΔG_*sol_polar*_) and non-polar solvation energy (ΔG_*sol_np*_).*

Clustering analysis was used to extract representative structures in simulations. Comparing the binding modes of T1551 and EPZ015666 with PRMT5 (**Figures 6c,d**), we could see that both inhibitors located in a hydrophobic pocket composed of Tyr304, Phe327, Ser578, and Phe580 when interacting with PRMT5. For EPZ015666, **Figure 6d** showed that its THIQ group formed strong cation–π interactions with partial positively charged methyl group of SAM. Actually, this feature has been reported as a key factor for EPZ015666’s efficiency in the previous study (Chan-Penebre et al., 2015). Compared with EPZ015666, although T1551 was lack of THIQ group, its phenyl ring in indole scaffold also formed cation–π interactions with SAM, explaining the inhibitory activity of T1551 against PRMT5 to some extent. Meanwhile, the pyrrole ring of indole group in T1551 formed π–π interactions with Phe327. T1551 also formed a hydrogen bond with the main-chain oxygen atom of Ser578. These together fasten the interactions of T1551 with PRMT5.

**FIGURE 7:**
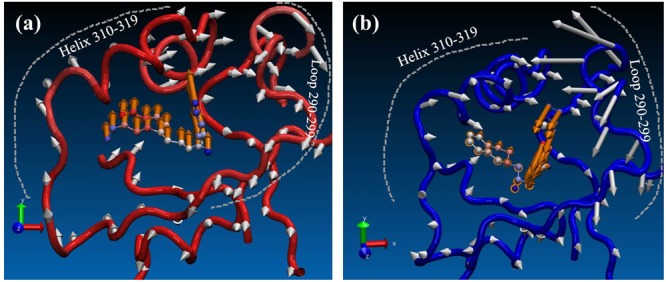
Comparison of effects of **(a)** T1551 and **(b)** EPZ015666 on the collective motion of PRMT5 in the simulation. The mode was obtained by normal mode analysis with Amber16 software and VMD NMWiz plugin. Only the normal modes of T1551 binding domain (10 Å around T1551) were shown here for clarity.

Finally, in order to see the effect of inhibitors on conformational space of PRMT5, normal mode analysis was carried out. For clear visualization, only the normal modes of T1551 binding domain (10 Å around T1551) were shown here. From **Figure 7**, it could be observed that the partial collective motion of EPZ015666 was opposite to that of T1551 during the simulation. As for PRMT5, the obvious differences in two complexes were reflected from helix residues 310–319 and loop residues 290–299. In the PRMT5-EPZ015666 system (**Figure 7b**), the helix and loop vibrated in the face–face direction, which seemed like to tighten the binding pocket and thus stabilize EPZ015666 into it. From **Figure 7b**, we also observed that the obviously higher amplitude motion of loop domain made major contributions in it. Nevertheless, in the PRMT5-T1551 system (**Figure 7a**), the helix and loop moved in the back–back direction, which led the pocket not as compact as that in PRMT5-EPZ015666 system. It may be closely associated with that EPZ015666 has better biological activity for PRMT5 than T1551.

## Discussion

PRMT5, as currently the only known type II PRMT, is also a member with the few inhibitors reported in PRMT family. As the relationship of PRMT5 and lung cancer is constantly revealed, it is urgent to search for effective inhibitors targeting PRMT5 for lung cancer therapy. SAM, as the natural substrate of PRMT5, is responsible for providing the methyl group in the process of methyl transfer. To date, most of PRMT5 inhibitors reported were aimed for SAM-binding site and designed to disturb the interaction of SAM and PRMT5 (Alinari et al., 2015; Smil et al., 2015; Mao et al., 2017). However, due to their native binding state, it is difficult to find small molecules with the inhibitory activity stronger than SAM. Fortunately, the discovery of EPZ015666 and its new binding site provides a new clue for developing non-SAM competitive inhibitors.

In this study, we identified T1551 as a non-SAM competitive PRMT5 inhibitor by virtual screening method. Subsequently, the anticancer activity of T1551 against NSCLC was studied from three aspects, namely PRMT5 methyltransferase activity, expression of target genes and signaling pathway mediated by target genes. For the former, the “on-target” and direct inhibitory effect of T1551 was reflected from the low PRMT5 enzymatic activity, and indirect effect was from the low expression level of PRMT5’s histone marker (H4R3me2s), which together suggested that T1551 inhibited PRMT5 methyltransferase activity.

For the latter, FGFR3 and eIF4E are two target genes of PRMT5 we focused here. We know that PI3K/AKT/mTOR pathway is a prototypic survival pathway in cancers, whose activation is closely related to cellular proliferation, growth, and mobility. FGFR3 promotes the survival of cancer cells just by stimulating the downstream PI3K/AKT/mTOR pathway (Kang et al., 2007; Hafner et al., 2010). Using RNA interference technology, Zhang et al. (2015) revealed that silencing PRMT5 could significantly downregulate FGFR3 and eIF4E expression. In our study, via inhibiting PRMT5, the identified T1551 was also shown to reduce the protein expressions of oncogene FGFR3 and eIF4E. Despite that the change of phosphorylated mTOR was not significant possibly due to the amplification effect of a signaling cascade, the concurrent reducing of phosphorylated AKT and ERK indicated that T1551 blocked the activation of PI3K/AKT/mTOR and ERK pathways in NSCLC cell line.

Previous studies emphasized that cation–π interaction between the tetrahydroisoquinoline group of EPZ015666 and partial positively charged methyl group of SAM was essential for EPZ015666’s higher competitive ability for PRMT5 relative to histone substrate (Chan-Penebre et al., 2015; Duncan et al., 2015). Replacing SAM with SAH, the binding affinity of EPZ015666 and PRMT5 could be decreased more than 100 times. Due to the importance of this feature, in the subsequently structural optimization of EPZ015666, cation–π has always been retained as a crucial interaction (Duncan et al., 2015). By comparing the binding modes of T1551 and EPZ015666 with PRMT5-SAM, we observed that the conformation of T1551 in PRMT5 new pocket was similar to that of EPZ015666. Importantly, the benzene ring of T1551 indole scaffold also formed strong cation–π interactions with the methyl group of SAM. This explains the inhibitory source of T1551 for PRMT5 to some extent.

In summary, a novel PRMT5 inhibitor T1551 with the indole scaffold was identified in this study, whose functional influence on PRMT5 was verified by a series of biological assays and theoretical inhibitory basis on PRMT5 was revealed by molecular dynamic simulation method. These results provide a lead compound for the further design of PRMT5 inhibitors, and contribute to the development of PRMT5-targeting cancer treatment.

## Author Contributions

XY, EL, and LL conceived the project. XY, EL, and QW designed the experiments. QW, JX, YL, JH, ZJ, and YW carried out the research and data analysis. XY, EL, LL, and QW wrote the paper.

## Conflict of Interest Statement

The authors declare that the research was conducted in the absence of any commercial or financial relationships that could be construed as a potential conflict of interest.
